# Podcasts as a teaching tool in orthopaedic surgery: Is it beneficial or more an exemption card from attending lectures?

**DOI:** 10.1007/s00132-020-03956-y

**Published:** 2020-08-04

**Authors:** Tobias Schöbel, Dirk Zajonz, Peter Melcher, Johannes Lange, Benjamin Fischer, Christoph-E. Heyde, Andreas Roth, Mohamed Ghanem

**Affiliations:** 1 Department of Orthopaedic Surgery, Traumatology and Plastic Surgery, University of Leipzig Medical Center, Liebigstr. 20, 04103 Leipzig, Germany; 2 ZESBO—Centre for Research on Musculoskeletal Systems, University of Leipzig, Semmelweisstr. 14, 04103 Leipzig, Germany; 3 Arbeitsgemeinschaft Lehre, Deutsche Gesellschaft für Orthopädie und Unfallchirurgie (DGOU), Straße des 17. Juni 106–108, 10623 Berlin, Germany

**Keywords:** Medical education, Educational technology, Surveys and questionnaires, Webcasts, Students, Medizinische Ausbildung, Bildungstechnologie, Umfragen und Fragebögen, Webcasts, Studierende

## Abstract

**Objective:**

The aim of this study was to evaluate the influence of the introduction of online podcasts as part of the main lecture series in orthopaedics on the number of lecture attendees, the examination results and the assessment of teaching by the students. Additionally, we evaluated the use of other media for examination preparation.

**Methodology:**

At the beginning and end of the lecture series questionnaires were handed out to the students to evaluate their attitudes towards attending lectures, the use of video podcasts and examination preparation. In addition, the number of lecture attendees and podcast usage during the semester were counted and the statements of the students in the evaluation assessments of orthopaedic teaching were evaluated. The examination results were correlated in a statistical analysis with the learning materials provided by the students for examination preparation.

**Results:**

At the end of the lecture series, 284 students stated that they used the lecture podcast about twice as often as attending lectures; however, for the majority of the students the provision of a video podcast was no reason not to attend the lecture. For example, 37.2% stated that they never and 26.8% stated that they rarely had not attended the lecture by providing the podcasts. Of the students 91–95% considered the availability of lecture podcasts to be a rather meaningful or very meaningful supplement to the lecture visit. Students increasingly used digital media to prepare for examinations instead of using traditional analogue methods. None of the learning methods or materials examined showed a statistically significant advantage in examination results.

**Conclusion:**

Students in the age of digitalization use a variety of learning materials and are no longer bound to classical analog teaching methods. The use of online podcasts had no negative impact on examination performance. Most students perceived lecture podcasts as a useful supplement to lecture attendance. The students praised the expansion of the teaching curriculum to include additional digital offers with positive comments in the evaluations, but without achieving an improvement in these student evaluations.

## Abstract (de)

**Zielsetzung:**

Ziel dieser Untersuchung war es, den Einfluss der Einführung von Online-Podcasts im Rahmen der Hauptvorlesungsreihe der Orthopädie auf die Anzahl an Vorlesungsbesuchern, die Prüfungsergebnisse oder die Bewertung der Lehre durch die Studierenden zu evaluieren. Zusätzlich wurde die Nutzung anderer Lehrmedien zur Prüfungsvorbereitung evaluiert.

**Methodik:**

Zu Beginn und zum Ende der Vorlesungsreihe wurden Fragebögen an die Studierenden ausgegeben, um deren Einstellung zum Vorlesungsbesuch, zur Nutzung von Video-Podcasts und zur Prüfungsvorbereitung zu evaluieren. Des Weiteren wurden die Vorlesungsbesucher und Podcast-Nutzungen während des Semesters gezählt und die Aussagen der Studierenden in den Evaluationsbewertungen der Lehre in der Orthopädie evaluiert. Die Prüfungsergebnisse wurde in einer statistischen Analyse mit den von den Studierenden angegeben Lernmaterialien zur Prüfungsvorbereitung korreliert.

**Ergebnisse:**

Am Ende der Vorlesungsreihe gaben 284 Studierende an, die Vorlesungs-Podcasts etwa doppelt so häufig genutzt zu haben wie die Möglichkeit zum Vorlesungsbesuch. Jedoch war die Bereitstellung eines Video-Podcasts für den Großteil der Studierenden kein Grund, die Vorlesung nicht zu besuchen. So gaben 37,2 % an „nie“ und 26,8 % „selten“ durch die Bereitstellung der Podcasts auf den Vorlesungsbesuch verzichtet zu haben. Für eine „eher sinnvolle“ oder „sehr sinnvolle“ Ergänzung zum Vorlesungsbesuch hielten 91–95 % der Studierenden die Verfügbarkeit von Vorlesungs-Podcasts. Die Studierenden verwendeten zur Prüfungsvorbereitung vermehrt digitale Medien anstatt klassischer analoger Methoden. Keine der überprüften Lernmethoden oder -materialien zeigte einen statistisch signifikanten Vorteil in der Prüfungsvorbereitung.

**Schlussfolgerung:**

Studierende im Zeitalter der Digitalisierung benutzen eine Vielzahl von Lernmaterialen und sind nicht mehr an klassische analoge Lehrmethoden gebunden. Der Einsatz von Online-Podcasts hatte keinen negativen Einfluss auf die Prüfungsleistung. Die meisten Studierenden empfinden Vorlesungs-Podcasts als eine sinnvolle Ergänzung zum Vorlesungsbesuch. Die Studierenden honorierten die Erweiterung des Lehrcurriculums um digitale Zusatzangebote mit positiven Kommentaren in den Evaluationen, allerdings ohne eine Verbesserung dieser studentischen Bewertungen zu erreichen.

## Background

Digitalization has an enormous impact on the world of working [2] and teaching [5] in the medical sector. The generation of medical students and doctors born since 1982 has grown up with information technology as an integral part of their lives [17]. Nevertheless, Internet-based teaching methods play a subordinate role in the training of young physicians [24]. The current range of digital teaching and learning formats in medical studies is heterogeneous and includes social communication tools, audio-based and video-based media, interactive formats and electronic examination systems [11].

A study conducted by the University of Leipzig in 2016 examined the influences of Internet-based media use in Germany. It was found that only 28% of the participants surveyed actively used the online media in classes. The main reasons given against the use of Internet-based media in classes were increased time expenditure and a lack of personnel support. Online media, such as interactive case studies, podcasts and subject-specific apps were rated as suitable for use in teaching. Social media such as Facebook and Twitter were rated as unsuitable for use in classes [24].

The recording of video podcasts in university teaching has been increasingly used since the beginning of the twenty-first century [11] either as an additional provision of flexibly retrievable learning materials [16], or as a teaching tool for direct feedback for students when learning clinical skills [10] or basics of medical communication [9, 10]. Video podcasts are also used as examination tools [9]. In general, the provision of video podcasts is perceived as positive by students [4–6, 8, 18]; however, the effectiveness of video podcasts as teaching tools remains controversial [5, 21, 22] as does the concern that the provision of online learning material will reduce the number of students attending optional classes [1, 13].

The effectiveness of video podcasts as teaching tools has been studied from different angles: Early studies concluded that a classical video podcast that combines the audio or video files recorded during a course with the lecturer’s slides can be as effective as traditional lectures [12, 23, 25]. Most studies on the influence of video podcasts on examination results found no significant influence of podcast use on examination results [3, 7, 21, 22]; however, previous studies suggested that the provision of video podcasts does not lead to a loss of lecture attendances [1, 13, 15].

In the winter semester 2018/2019, video podcasts of lectures were recorded for the first time in the main lecture series of orthopaedic medicine at a Central German university and made available to students in a password-protected area (student portal). The aim of this study was to determine the influence of this change in teaching curriculum on examination results, attendance at lectures and the evaluation of courses by students.

Specifically, the study aimed at answering the following questions:

1. Is the provision of video podcasts a reason for students not to attend more or less frequently the facultative lectures of orthopaedics?
2. Which media do the students use to prepare for the examination and when?
3. Does the use of video podcasts have any influence on the examination results of individual students?

Additionally, the use of media preferred by students for examination preparation was analysed to classify the role of lectures in the context of teaching media in times of digitalization. We hope to be able to make recommendations for teaching in orthopaedics and trauma surgery on the basis of the results.

## Methodology

### Technical implementation

The video podcasts were a classical combination of the lecturers’ slides in Powerpoint™ format (Microsoft, Seattle, WA, USA) and simultaneous audio recording by Camtasia Studio 9 (Techsmith®, Okemos, MI, USA) of the lecture. Of the 26 lectures held 21 were recorded as podcasts.

The video podcasts were made available in the password-protected student portal of the medical faculty at the end of the lecture week and were activated for all students of the ninth semester of human medicine until the end of the winter semester 2018/2019. In addition to the 21 lectures, the lecture slides were provided in PDF format (Adobe Inc., San Jose, CA, USA) before or after the respective lecture.

### Data collection

In the winter semester 2018/2019 there were 255 students enrolled in the ninth semester of human medicine, which includes the orthopaedic lectures and examination. Additionally, 51 students of other study semesters participated in the obligatory final examination of orthopaedics and were able to attend the lectures or use the video podcasts.

**Fig. 1:**
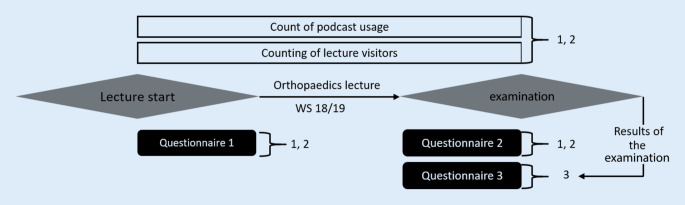
Data collection in the winter semester (WS) 2018/2019. *1* Influence of online podcasts on lecture attendees. *2* Use of media for examination preparation. *3* Influence of podcasts and other media on the examination result

The data were determined by means of three questionnaires, the continuous counting of lecture attendees, the recording of the number of podcasts retrieved from the student portal, the evaluation of the examination results and the evaluation of the student evaluations of the orthopaedic lecture series over the past 3 years (Fig. 1).

The number of attendees was counted at the beginning of each lecture. The system of the student portal enables retrieval of the video podcast to be recorded as well as the time of the retrieval.

The survey on students’ motivation to attend lectures, their attitudes towards the use of video podcasts in teaching, their preferred learning materials and their preparation time for the final examination was carried out in an anonymous questionnaire at the beginning and end of the lecture series and evaluated with EvaSys (Electric Paper Evaluationssysteme GmbH, Lüneburg, Germany).

The first questionnaire was answered at the beginning of the first facultative lecture in the form of a sample of 85 students. It was divided into:

- 3 questions about the previous attendance of lectures and the intention to attend lectures in the upcoming winter semester
- 4 questions about experiences and settings for video podcasts
- 1 free text field for any comments on the use of podcasts in student teaching.

The second questionnaire was distributed at the end of the lecture series as part of a compulsory course and completed by 284 students. It was divided into:

- 2 questions about the actual attendance of lectures in the past winter semester
- 4 questions about students attitude towards video-podcasts
- 2 questions about the possible waiver of the lecture visit by the provision of video podcasts of the lectures and, if applicable, the reasons
- 7 questions about the time of examination preparation and the preferred learning materials (multiple choice).

A third questionnaire was also handed out before the compulsory examination and the student’s matriculation number could be voluntarily provided. The results of this questionnaire could then be correlated with the examination results. The questionnaire was completed by 225 students. It was divided into:

- 2 questions about setting up video podcasts
- 1 question about attending lectures in orthopaedics last winter semester
- 1 question about the preferred learning materials for examination preparation.

### Statistical analysis

The data obtained in the third questionnaire were correlated with the examination results of the students using the Spearman rank test. The program SPSS (IBM Corp. Released 2016. IBM SPSS Statistics for Windows, Version 24.0. Armonk, NY, USA) was used and the significance level was fixed at *p* < 0.05.

### Data protection

All participating lecturers had consented in writing to the publication of the lecture slides as podcasts. No patient data were published in the lectures. All students completed a declaration of consent to the use of anonymized data, in particular examination results, as part of the survey. In the absence of consent or rejection, the student was not included in the analysis.

## Results

### Influence of video podcasts on lecture attendance

**Fig. 2:**
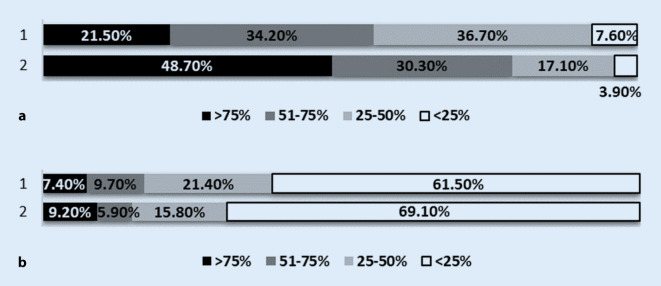
**a** Intention of students to attend lectures at the beginning of the lectures: *1* how many lectures will you attend this semester (of the total number of lectures)? (*n* = 79 out of 85 respondents). *2* How many lectures of orthopaedics do you want to attend this semester (of the total number of lectures of orthopaedics)? (*n* = 76 out of 85 respondents). **b** Self-declaration of the attendance of lectures by the students at the end of the semester: *1* how many lectures did you attend this semester (of the total number of lectures)? (*n* = 272 out of 284 respondents). *2* How many lectures of orthopaedics did you attend this semester (of the total number of lectures of orthopaedics)? (*n* = 272 out of 284 respondents)

**Fig. 3:**
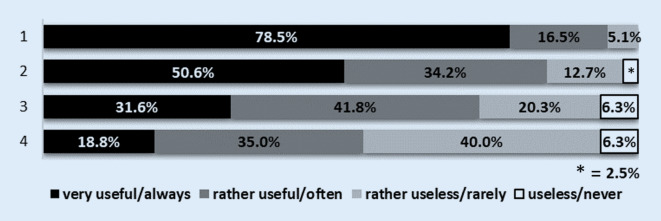
Assessment of podcast usage by students at the beginning of the lecture series. *1* Do you consider the provision of a podcast to be a useful supplement to attending lectures? (*n* = 79 out of 85 respondents). *2* Do you see the provision of a podcast as a useful alternative to attending lectures? (*n* = 79 out of 85 respondents). *3* Have you ever used podcasts offered for teaching purposes in the past as a supplement to attending lectures? (*n* = 79 out of 85 respondents). *4* Have you ever used podcasts offered in the past as an alternative to attending lectures? (*n* = 80 out of 85 respondents)

**Fig. 4:**
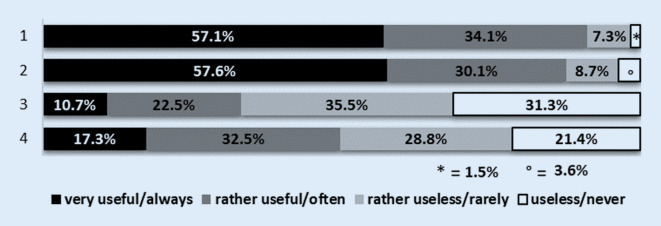
Assessment of podcast usage by students at the end of the lecture series. *1* Do you consider the provision of a podcast in the orthopaedics lecture to be a useful supplement to attending the lecture? (*n* = 273 out of 284 respondents). *2* Do you see the provision of a podcast in the orthopaedics lecture as a useful alternative to attending the lecture? (*n* = 276 out of 284 respondents). *3* How often have you used the podcasts offered in orthopaedics as a supplement to attending lectures? (*n* = 262 out of 284 respondents). *4* How often have you used the podcasts offered in orthopaedics as an alternative to attending lectures? (*n* = 271 out of 284 respondents)

**Fig. 5:**
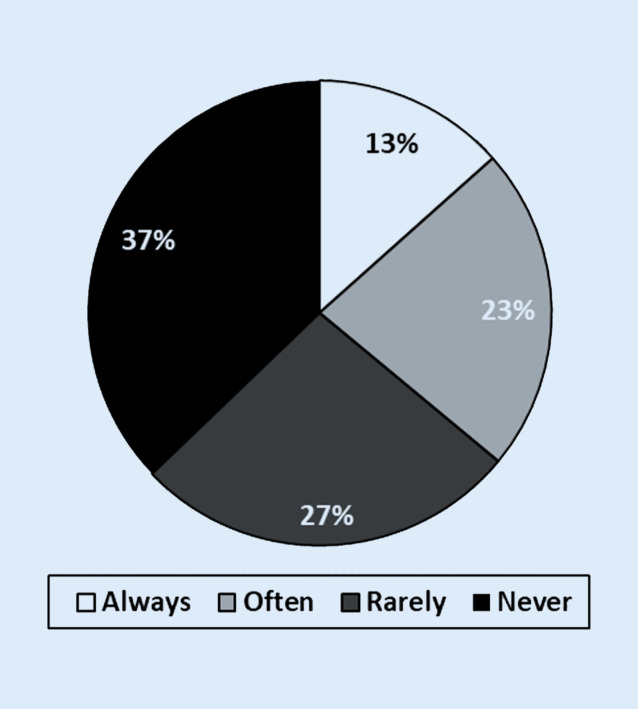
Students’ assessment of the question: how often have you avoided attending as a result of podcast provision?

Of the students surveyed at the beginning of the lecture period (questionnaire 1) 79% stated that they would like to attend more than half of the orthopaedics lectures (Fig. 2a). In the survey at the end of the lecture series (questionnaire 2) only 15.1% of the students stated that they had attended more than 50% of the lectures in orthopaedics (Fig. 2b). At the beginning of the lecture series 95% of the students stated that the provision of a podcast was a useful supplement to attending the lectures and 84.8% of the interviewed students found the provision of a video podcast of the lecture to be a useful alternative to attending lectures (Fig. 3). At the end of the lecture series 91.2% of the respondents considered the video podcasts to be a meaningful supplement to attending lectures and 87.7% a meaningful alternative to attending lectures (Fig. 4). At the end of the lecture series one third of the students surveyed stated that they had used the podcasts in orthopaedics as a supplement to their attendance at the lectures, and half of the students stated that they had used the podcasts as an alternative to their attendance at the lectures (Fig. 4). Of the students 37.2% stated that they never and 26.8% stated that they rarely did not attend lectures as a result of podcast provision (Fig. 5).

**Fig. 6:**
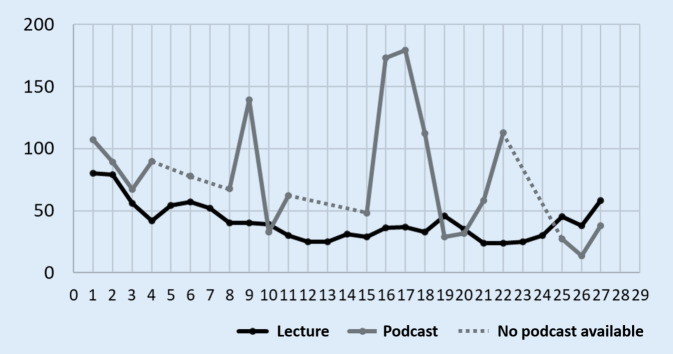
Number of students present in the lecture and number of accesses to the respective lecture podcasts. X-axis: number of lecture and y-axis: number of students

The lecture series had an average of 44 visitors per lecture and the podcasts had an average of 78 hits per lecture (Fig. 6). Lectures that were recorded as podcasts, but for which no lecture slides were provided in PDF format, received the highest number of hits. Most lecture visitors were counted at the beginning and end of the lecture series (Fig. 6).

### Media use for examination preparation

**Fig. 7:**
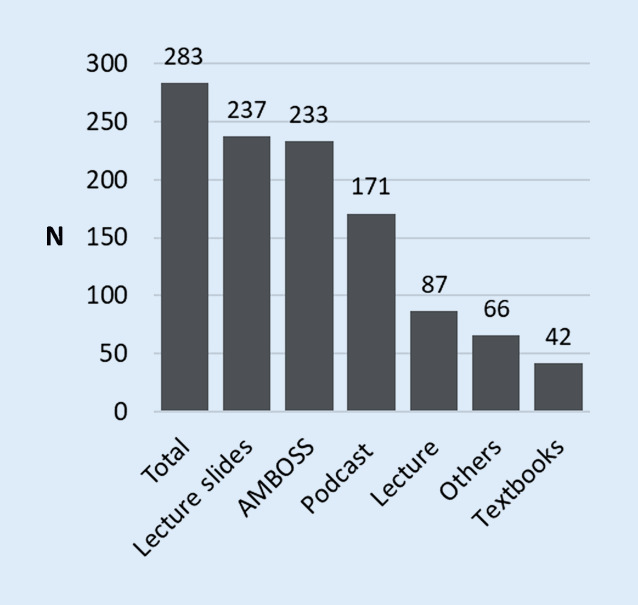
Number of students who prepared for the examination with a specific learning material (multiple answers possible, *n* = 283 out of 284 respondents)

With 83.7%, the most popular learning material for examination preparation among the students surveyed was the lecture slides provided by the lecturers in PDF format. This was immediately followed by the online learning tool AMBOSS (AMBOSS GmbH, Cologne, Germany), which was used by 82.3% of the students for examination preparation. Of the students 60.4% stated that they had prepared for the examination with the podcasts of the lecture and 30% of the students stated that they attended the classical lecture to prepare for the examination. Other learning materials (23.3%) and textbooks (14.8%) were only used by a few students for examination preparation (Fig. 7).

Regardless of the learning material 45% of the students stated that they prepared for the examination in the week before the examination. An exception was the preparation for the examination by attending the lectures: one third of the attendees stated that they were preparing for the final examination earlier at the beginning of the lecture series. The students surveyed stated that they tended to use the media regularly during the lecture series rather than to prepare themselves in irregular intervals for the examination.

**Fig. 8:**
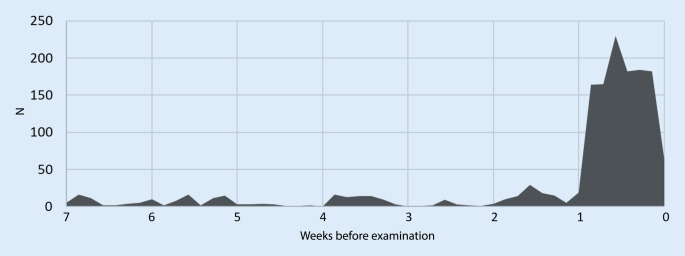
X‑axis: weeks to exam and y‑axis: number of accesses to lectures from the student portal

The access figures from the student portal showed that 78.1% of the accesses to the podcasts took place in the week before the examination. In the first week of the lecture series 3% of the accesses to the lecture podcasts took place (Fig. 8).

### Influence of podcasts and other media on the examination results

The examination consisted of 30 multiple choice questions with selection of a single answer. The final examination was passed with a minimum of 18 correctly answered questions. The grades were awarded in accordance with the study regulations of the medical faculty. The questions were prepared by the lecturers and the students had 45 min to answer the examination questions. The students achieved an average grade of 1.5 and all candidate passed the final exam. Of the students 184 achieved grade 1 (≥90% correct answers to questions), 95 students grade 2 (≥80%) and 27 students grade 3 (≥70%).

**Fig. 9:**
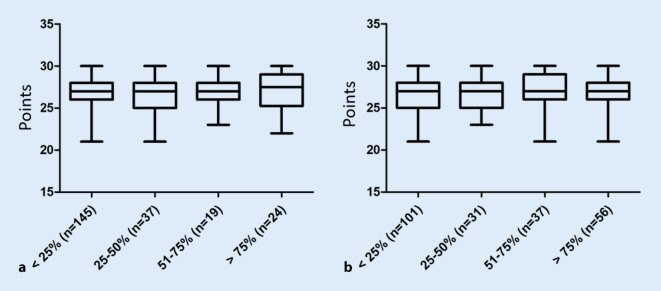
Boxplots of the achieved points in the final examination (Y-axis) depending on **a** the frequency of the students’ attendance at the lectures (X-axis) and **b** the frequency of the use of an online podcast as an alternative to the attendance at the lectures (X-axis)

There was no statistically significant correlation between podcast use and the examination results of the students (r = 0.06848; *p* = 0.3064). Additionally, there was no statistically significant correlation between the examination results and examination preparation through attendance at lectures (r = 0.05644; *p* = 0.3995), use of lecture slides (r = −0.01413; *p* = 0.8331), textbooks (r = −0.04728; *p* = 0.4804), AMBOSS (r = −0.08483; *p* = 0.2049) or other learning materials (r = −0.02999; *p* = 0.6553). Nor did the frequency of attending lectures (r = 0.02346; *p* = 0.7264) (Fig. 9a) or the frequency of podcast use as an alternative to attending lectures (r = 0.06797; *p* = 0.3101) (Fig. 9b) show any correlation to the examination results.

## Discussion

At the beginning of the lecture series approximately 80% of a sample of 85 students (questionnaire 1) wanted to attend more than half of the orthopaedics lectures (Fig. 2a); however, only 15.1% of the students surveyed at the end of the lecture series (questionnaire 2) stated that they had attended more than 50% of the orthopaedics lectures (Fig. 2b). This self-assessment is in line with our count of the number of lecture attendees during the lecture series, which was 14.4% with an average of 44 attendees to the lectures out of 306 students surveyed. We assume that the discrepancy between the declaration of intent of the students and the actual attendance of the lecture results from our research setting: The first questionnaire was handed out during the facultative first lecture, which was presumably attended by students who were more interested in the subject and had an affinity for the lecture. The second questionnaire was handed out before the obligatory final orthopaedics examination, in which all students for the corresponding semester had to participate.

The video podcasts were used about twice as often as the lecture visit for examination preparation (Fig. 7), this assessment of the students was confirmed by our counts of lecture attendees and podcast users (Fig. 6). It should be noted that the use of the podcast offer does not exclude attending lectures: 10.7% of the students surveyed stated that they had always used the podcast and 22.5% frequently as a supplement to attending lectures (Fig. 4). Additionally, based on our survey design it could not be excluded that some students watched individual podcasts more than one time or did not watch the podcasts completely.

Only 13% of the students surveyed answered the concrete question of how often they had given up attending lectures due to the provision of a podcasts, with always, while 23% said they had done so frequently (Fig. 5). This observation coincides with previous studies which suggest that the provision of video podcasts does not lead to a heavy loss of lecture visits [1, 13, 15].

Mattick et al. compared the number of students attending facultative telemedical courses and courses with a lecturer present in 2003/2004. They found no statistically significant difference between the numbers of participants for both groups. Interestingly, Mattick et al. also described the trend observed by us that as courses progressed, fewer students participated in facultative courses with a lecturer present [13]. Later, during a 3-year observation period (2004–2006), Billings-Gagliardi and Mazor observed the participation behaviour of students at the University of Massachusetts Medical School, with at least 95% of the optional courses recorded and the material made available online for viewing or downloading. In an open question 197 students justified their decision to attend individual courses or to stay away from them. Of these students 90% stated that the availability of electronic learning materials had no influence on their decision to attend or stay away from events. The remaining 10% indicated that the availability of electronic teaching materials had contributed more to participation in the courses concerned [1]. These results are consistent with our observations that 91–95% of students consider the availability of lecture podcasts to be a meaningful complement to lecture attendance (Figs. 3 and 4).

According to Kuhn et al. almost all medical students [11] make intensive use of classical digital media such as PDF files and PowerPoint® presentations. At the same time, these classical digital media represent the only format that is established throughout Germany as part of medical studies [19]. In addition, a large number of students now use web-based knowledge and examination platforms for efficient examination preparation [11]. This statement is in line with our findings, showing a clear preference of the students for examination preparation with digitally available learning materials, such as lecture slides, web-based knowledge and examination platforms (AMBOSS) and video podcasts, coincides with classical analogue learning options through attendance at lectures or textbooks (Fig. 7).

There are several studies on the influence of video podcasts on medical examination results [3, 7, 21, 22]. Brockfeld et al. compared the results of 205 students in a 301 MC questionnaire in 2014, where 1 group followed a 41‑h course with a lecturer present and the other group followed the 41‑h course as a video. The first group answered 78.3% of the questions in the subsequent MC test correctly, the second group 78.6% [3]. In our study, those students who used podcasts to prepare for the final examination answered 89.8% of the questions correctly on average, and those who prepared without podcasts answered 89.0% correctly on average. Schreiber et al. came to similar conclusions: 100 students were randomized to attend either a lecture with a lecturer present or watch a video podcast on two clinical topics and then examined both groups with a MC questionnaire. The lecture groups answered 90.2% of the questions correctly on average, the podcast groups 87.8% without being able to find a statistically significant difference [21]. Davis et al. also found no significant difference in the increase in knowledge between a lecture group and a podcast group on the same medical topic in a similarly designed experimental setup with 229 medical students [7]. An exception is the study by McNulty et al. in which the frequency of podcast use by students was correlated with their course grades: Interestingly, the group of students who had used the fewest video podcasts for preparation had significantly better results than the group of students who had used the video podcast most intensively [15]. The authors justified their observation with the fact that the frequency of use of video podcasts seemed to be higher among students who had problems understanding the topic. Another study by the same research group showed that more frequent use of Internet-based learning materials by students was associated with better examination results [14]. In a study by Rizzolo et al. those students in the examinations who relied the least on Internet-based media for examination preparation scored worst [20].

In the final evaluation of the orthopaedics lecture series, the students gave positive feedback throughout for the provision of online podcasts; however the thematic overlapping of lecture topics, an overloading of individual topics and a missing common theme during the lecture series were criticized. The statements of our students in the free text responses corresponded with the observations of Schreiber et al. who in their previously described experimental set-up had the students evaluate both the quality of the lecture and the online podcasts. The presentation and content quality of the podcasts were consistently rated somewhat worse than that of the lectures; however, the students praised the possibility of flexible learning over time with an online podcast and the ability to pause the podcast, repeat specific sections or the whole podcast [21].

Ultimately, the quality of the lectures in terms of content, an interdisciplinary structure and the lecturers’ lecturing skills seem to have been decisive for the students’ assessment.

From the perspective of the lecturers, a critical aspect of the provision of online podcasts as part of a lecture series which has to be discussed, are data protection aspects. The use of patient-related data and images must be made anonymously and with the written consent of the parties involved. Patients participating in the lectures for demonstration purposes must sign a consent form or, if necessary, be removed from the lecture transcript. A lack of personnel and technical support [24], uncertainties in dealing with legal aspects of the online availability of lecture materials and the associated increased workload are still existing obstacles in the digitalization of such courses.

## Limitations

A limitation of our investigation is that not all lectures in orthopaedics could be provided as podcasts and that not all lectures were provided with PDF lecture slides. A conclusion as to whether the absence of individual podcasts or lecture slides had an influence on the relevant lecture topic in the examination results was not possible due to the test setting.

Furthermore, the average very good results in the final examination make it more difficult to investigate the influence of the learning materials or learning times used on the examination results. It might have been possible to find correlations between these influencing factors and the individual examination results if there had been a greater disparity.

Based on the given data, another limitation lies in the fact that no exact comparison between the changes of students’ motivation to participate in a lecture was possible. The first questionnaire was answered during the facultative first lecture with possibly more interested students whereas the second and third questionnaires were taken before the compulsory examination, with all students of the semester participating. This possibly caused a bias and increased the difficulties in comparing the data. A follow-up of all questionnaires was not possible due to the anonymous data collection for the first and second questionnaires.

Finally, in order to determine whether the provision of video podcasts would reduce the number of students attending the lectures, it would be desirable to count the number of attendees in the previous years of the orthopaedics lectures in order to objectify the statements made by the students.

## Conclusion

Students in the age of digitalization use a variety of learning materials and are no longer tied to classical analogue teaching methods. They are increasingly using digital media such as online lecture slides, web-based knowledge and examination platforms and online podcasts to prepare for examinations instead of traditional analogue methods, such as attending lectures or acquiring knowledge through textbooks. The use of online podcasts did not have a negative or positive impact on the test performance.

The provision of video podcasts of the main lectures in orthopaedics was no reason for the majority of students not to attend the lecture. For most students, lecture podcasts were a useful supplement to attending lectures, and students praised the expansion of the teaching curriculum to include additional digital offers with positive comments in the evaluations.

## Abbreviations

MC: Multiple choice
WS: Winter semester
